# Building block and rapid synthesis of catecholamines-inorganic nanoflowers with their peroxidase-mimicking and antimicrobial activities

**DOI:** 10.1038/s41598-020-59699-5

**Published:** 2020-02-19

**Authors:** Cagla Celik, Nilay Ildiz, Ismail Ocsoy

**Affiliations:** 1 Department of Analytical Chemistry, Faculty of Pharmacy, Erciyes University, 38039 Kayseri, Turkey; 2 Department of Pharmaceutical Microbiology, Faculty of Pharmacy, Erciyes University, 38039 Kayseri, Turkey

**Keywords:** Biocatalysis, Biotechnology, Chemistry, Materials science, Nanoscience and technology

## Abstract

Protein incorporated flower-shaped hybrid nanostructures have received highly considerable attention due to their greatly enhanced catalytic activities and stabilities. Up to date, proteins, enzymes (mostly considered as proteins), and amino acids (as the building blocks of peptides and proteins) have been used as organic components of the hybrid nanoflowers. Herein, we present a rational strategy to rapidly form catecholamines (dopamine, epinephrine and norepinephrine)-copper ion (Cu^2+^) incorporated nanoflowers (cNFs) mostly in 3 hours and show their peroxidase-mimic catalytic, dye degradation and antimicrobial activities through Fenton-like reaction mechanism. We systematically studied effects of experimental parameters including catecholamine concentrations, reaction time and reaction pH values, on formation of the cNFs. We also explained that norepinephrine nanoflower (neNF) with its porous structure, high surface area, polar surface property behaves as an efficient Fenton agent by exhibiting highly much catalytic activities compared to dopamine nanoflower (dNF) and epinephrine nanoflower (epNF). We claim that the NFs formed using nonprotein molecules can be used in designing new generation nanobiocatalytics, antimicrobial agents, nanobiosensors and pharmaceutical products.

## Introduction

Many conventional methods, chemical modification and immobilization, have been developed with both aim of solving instability problem of free enzymes in aqueous solution and increasing their catalytic activities^1–9^. Despite great performance spent on these methods, only high stability so far was accomplished with modified enzymes. Unfortunately, modified enzymes have not provided any increase in activities compared to free enzymes and even loss of activity was observed in some reported works^8–10^. It is worthy to mention that while limited mobility of enzymes in immobilized form may increase their stabilities, it adversely affects catalytic activities of enzymes owing to their unfavourable conformation and serious mass transfer limitations occurred between enzymes and substrates^10–12^.

To address these disadvantages of modified or immobilized enzymes, fabrication of hybrid organic–inorganic nanoflowers (NFs) with greatly enhanced catalytic activities and stabilities were discovered by Zare and co-workers^13,14^. In this elegant *in situ* immobilization approach, available amine groups of enzymes preferentially react with Cu^2+^ in phosphate buffered saline (PBS) for hierarchical and self-assembled formation of enzymes-copper phosphate (Cu_3_(PO_4_)_2_) hybrid NFs in 72 hours (hrs). However, almost all reported studies on NFs rely on use of proteins and enzymes as organic components. In subsequent studies, various single and multi-enzymes have been utilized to design novel nano-biocatalytic systems or nano-biosensors used for applications in biomedicine, biocatalysis and bioanalytical sciences. For instance, researchers used different of types commercially available or isolated single enzymes for production of NFs by following and modifying reported method for showing their greatly enhanced enzymatic activities towards model substrates or targets (hydrogen peroxide, dopamine, m-cresol, phenol, etc)^14–24^. Additionally, Mao *et al*., and Avidad *et al*., successfully combined glucose oxidase (GOx) and horseradish peroxidase (HRP) enzymes in one single NF system for developing colorimetric sensor and 3D microfluidic paper-based device, respectively^19,25^. Using dual enzymes in NF provided cascade enzymatic reactions. Furthermore, whole plant extracts were used as organic components by Ildiz *et al* and Baldemir *et al* to form novel organic-inorganic NFs with an intrinsic peroxidase-like activities. However, the corresponding molecules in whole extracts acted as organic components for NF formation were not identified yet^26,27^. Herein, we present, for the first time, synthesis of novel organic–inorganic nanoflowers (NFs) almost in 3 hrs using catecholamines (dopamine, epinephrine and norepinephrine) as organic components and Cu^2+^ ions as inorganic components. We also elucidate effects of experimental parameters on formation of cNFs with explanations of their catalytic, dye degradation and antimicrobial activities depending upon Fenton-like reaction mechanism.

## Materials

All catecholamines (dopamine, epinephrine and norepinephrine), 2,2′-azino-bis (3-ethylbenzothiazoline-6-sulfonic acid)-diammonium salt (ABTS), thiazolyl blue tetrazolium bromide, hydrogen peroxide (H_2_O_2_), Copper (II) sulfate pentahydrate, (CuSO_4_·5H_2_O), salt precursors for PBS (NaCl, KCl, Na_2_HPO_4_, KH_2_PO_4_, CaCl_2_.2H_2_O, MgCl_2_.6H_2_O) were purchased from Sigma-Aldrich. For all experiments, ultrapure water with 18.2 MΩ (Millipore Co., USA) was consumed. *E. coli* ATCC 35218 (Gram negative) and *S. aureus* ATCC 25923 (Gram positive) used as bacterial strains, and *C. albicans* ATCC 10231 used as fungi were obtained from Erciyes University Pharmaceutical Microbiology Laboratory collection.

## Methods

UV-Vis Spectroscopy (Shimadzu 1800) was used for determination of peroxidase like activities of the cNFs. For imaging morphologies of cNFs, Scanning Electron Microscopy (SEM, ZEISS EVO LS10) was operated. Simply, powder of the cNFs was deposited on SEM stubs, then each stub was coated with gold (Au) using sputter coating device prior to SEM operation. The Energy Dispersive X-Ray Analysis and Mapping (EDX) equipped to SEM were used for presence of copper metal and other elements (carbon, oxygen, nitrogen and phosphorus) in the cNFs. The bonds stretching and bending in the cNFs were characterized by both of Fourier Transform Infrared (FTIR) and RAMAN Spectroscopies. The crystal structure of the cNFs with peak positions of Cu_3_(PO_4_)_2_ primary crystal was analyzed X-Ray Diffraction (XRD, Bruker AXS D8 Advance Model). Brunauer-Emmett-Teller (BET) was used for analysis of surface area of the cNFs.

### Synthesis of catecholamines nanoflowers

For synthesis of the cNFs, reported methods were modified and followed^13,14^. Firstly, different concentrations of catecholamines (dopamine, epinephrine and norepinephrine) as organic parts and 120 mM of stock Cu^2+^ solution as an inorganic part were freshly prepared in ultrapure water prior to mixing them in PBS solution for formation of the cNFs, respectively. Each catecholamine solution was added into 10 mM PBS (pH 7.4) and followed by addition of Cu^2+^ solution. Before incubation of the resulting mixture under undisturbed condition, each mixture was vigorously shaken or vortexed to increase the interaction between catecholamines and Cu^2+^ ions, which is considered as a necessary step for homogeneous nucleation and uniform cNFs formation. It is noted that final concentrations of catecholamines and Cu^2+^ ions were adjusted to 0.02 mg/mL and 0.8 mM, respectively. The cNFs formation, especially for dNF, was observed in 3 hrs at 25 °C.

Although formation of cNFs was examined based on concentrations of dopamine, epinephrine and norepinephrine molecules, morphologies of the dNFs were investigated as a function of reaction time, pH values of PBS solutions, type of metal ions and post addition of dopamine molecules on pre-formed Cu_3_(PO_4_)_2_ primary crystals owing to rapid and ideal formation of the dNFs.

### Catalytic activities of catecholamines nanoflowers against model substrate and dye

The catalytic activities of each catecholamine nanoflower (cNF) (10 μg/ml) were tested based on oxidation of ABTS (1 mM) and thiazolyl blue tetrazolium bromide (0.5 mM) used as a model substrate and dye, respectively in the presence of 22.5 mM H_2_O_2_. All catalytic reactions were completed in 10 mM PBS solutions (pH 4.0). The changes in absorbance values based of production of ABTS^•+^ and formazan type derivative were recorded at 417 nm and 570 nm, respectively using a UV-Vis spectrophotometer.

### Antimicrobial activities of catecholamines nanoflowers

The neNF was selected for all antimicrobial experiment due to much catalytic activity among other cNFs. The antimicrobial activities of neNF towards *E. coli* ATCC 35218 (Gram negative) and *S. aureus* ATCC 25923 (Gram positive) used as model bacterial strains, and *C. albicans* ATCC 10231 used as a model fungi were performed based upon Clinical Laboratory Standards Institute (CLSI) guidelines via broth microdilution method^28–31^.

In typical antimicrobial measurement procedure, the bacterial cells were prepared by culturing them in Mueller Hinton broth and incubated at 37 °C for 14 hrs to fix the bacteria cultures to 0.5 McFarland. Based on broth microdilution method, bacterial and fungi cell solutions, CuSO_4_, norepinephrine and neNFs were separately mixed in 96-well microtiter plates and each mixture was incubated at 37 °C for 18–24 hrs for bacteria and 48–72 hrs for fungi to examine microbial growth. All experiments were carried out in triplicate.

## Results and Discussion

In nanoflower (NF) synthesis, nitrogen atoms of the amine groups in catecholamines reacted with Cu^2+^ ions in PBS solution to form catecholamine-(Cu_3_(PO_4_)_2_) primary crystals as seeds in nucleation step. These primary crystals provide multi-nucleation sites for anisotropic growth in process nanoflower formation. In the growth step, continuous feeding of primary crystal with catecholamines led to occurrence of large petals containing catecholamine-(Cu_3_(PO_4_)_2_), then catecholamines in petals functioned as adhesive molecules to bind the petals each other. In the last step, combination of the petals was completed with saturation of anisotropic growth for the formation of the whole and single nanoflowers.

**Figure 1:**
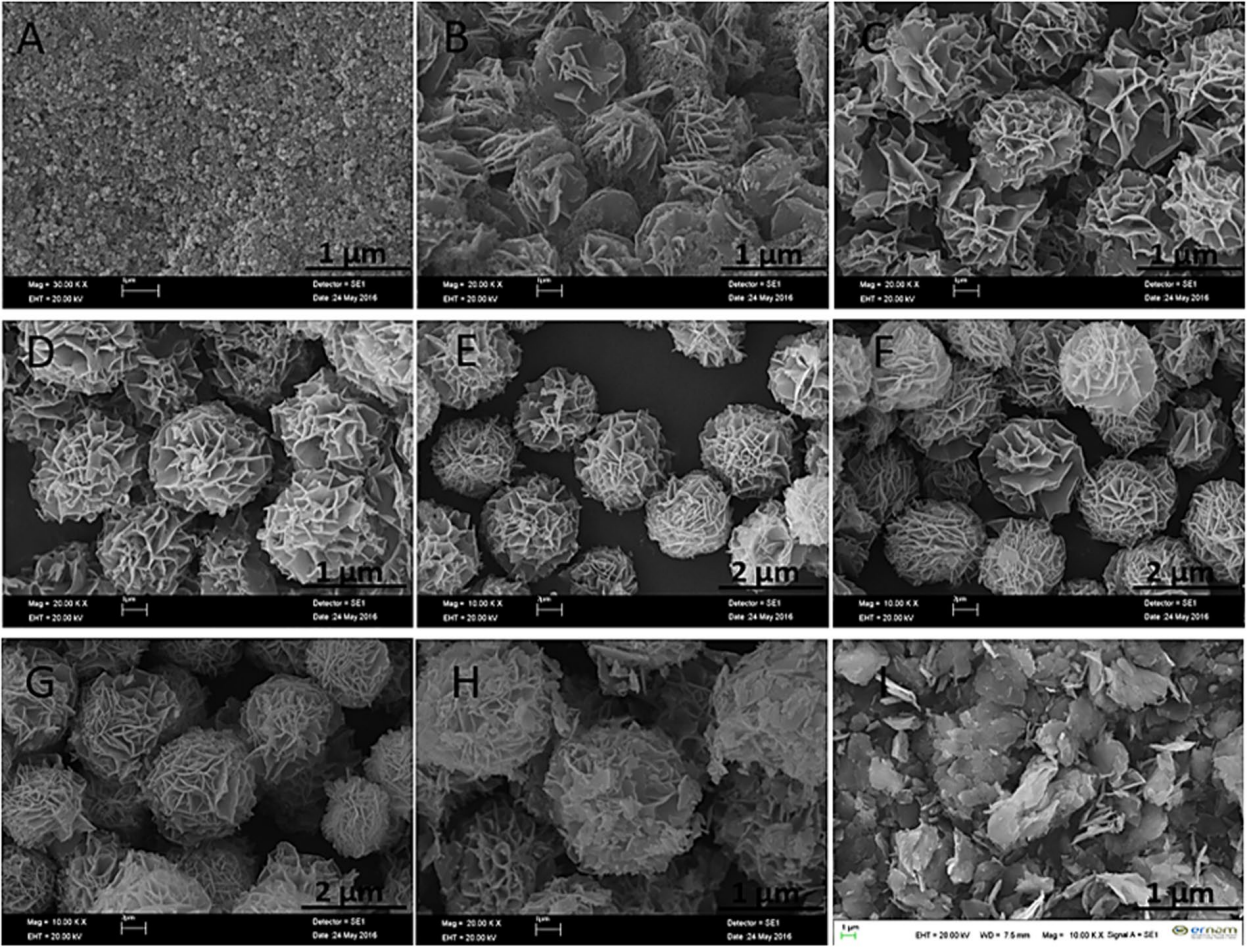
SEM images of dNFs using (0.02 mg/mL) dopamine and Cu^2+^ (0.8 mM) formed in different reaction times. (**A**) 1 hr, (**B**) 3 hrs, (**C**) 6 hrs, (**D**) 12 hrs, (**E**) 24 hrs, (**F**) 48 hrs, (**G**) 72 hrs and (**H**) 96 hrs. (**I**) SEM image of Cu_3_(PO_4_)_2_ primary crystals formed in 72 hrs without dopamine.

There common catecholamines (dopamine, epinephrine and norepinephrine) were used as organic parts to form catecholamines-(Cu_3_(PO_4_)_2_) (cNFs). The formation of dopamine nanoflower (dNF) using (0.02 mg/mL) dopamine and Cu^2+^ (0.8 mM) were studied based on different reaction times (1 hr, 3 hrs, 6 hrs, 12 hrs, 24 hrs, 48 hrs, 72 hrs and 96 hrs) as shown in Fig. 1. Although typical protein/enzyme-inorganic nanoflower formed in 72 hrs, almost all dNF was rapidly formed in 3 hrs. In 1^st^ hrs of incubation, only formation of spherical seeds was observed (Fig. 1A). Figure 1B shows an elegant example of the building block formation of dNFs from dopamine-Cu_3_(PO_4_)_2_ primary crystals through the bottom-up approach. It also demonstrates that binding of petals together and growth process were almost completed in 3 hrs. In general, NFs using various organic components (proteins, enzymes, amino acids, plant extracts and standard plant molecules) and Cu^2+^ ions as inorganic components were self-assembly formed in 72 hrs by giving blue-colored precipitates (considered as indication of organic molecule-Cu_3_(PO_4_)_2_ formation). In contrast to that, two main potential mechanisms, dopamine-Cu_3_(PO_4_)_2_ formation and oxidation process between dopamine molecules (as like polydopamine formation process), may simultaneously contribute formation of dNFs. We claim that obtaining black-colored precipitate of the dNFs was attributed by dopamine-Cu_3_(PO_4_)_2_ formation and oxidation of dopamine or partially formation of polydopamine. With 6^th^ hrs of incubation, dNF formation was completely carried out as seen in Fig. 1C. In addition to that no remarkable difference in size and shape of dNFs formed in 12^th^ hrs, 24^th^ hrs, 48^th^ hrs, 72^nd^ hrs and 96^th^ hrs of incubations as presented with SEM in Fig. 1D–H, respectively. Figure 1I presents that while large and separate crystals were observed, but no NFs were obtained without the dopamine molecule, which proves the adhesive role of dopamine molecules in binding of the petals together for eventual dNF formation.

**Figure 2:**
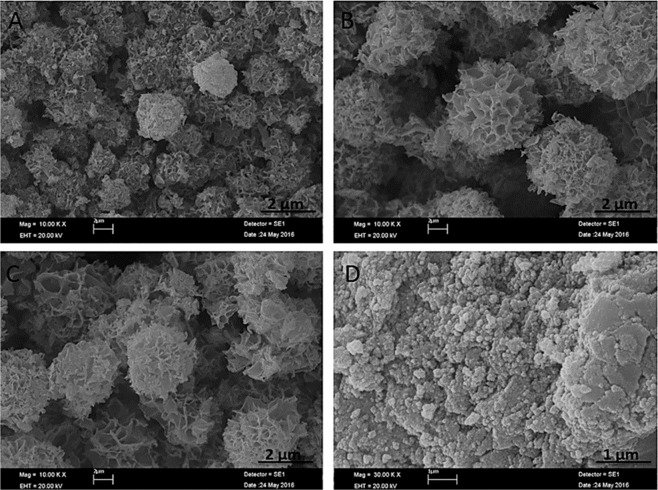
SEM images of dNFs formed in pH of (**A**) 5, (**B**) 6, (**C**) 9 and (**D**) 10.

We claim that the dNFs with uniform and ideal flower shaped were produced at pH 7.4 of 10 mM PBS solution. The formation of dNFs were evaluated at various pH values of PBS solutions (pH 5, 6, 9 and 10). The dopamine, as a positively charged molecule, was not involved in NF formation owing to strong positive repulsion between dopamine molecules and Cu^2+^ ions at pH < 5 (data not shown here). Although dopamine molecules are still highly positively charged at pH 5, shape of the dNF was apparently splayed owing to loosely binding of the petal (Fig. 2A), More uniform morphology was obtained when dNF was synthesized at pH 6 (Fig. 2B). The charge of dopamine molecule is expected to be close to neutral at pH 9, then dNF was obtained with distorted morphology (Fig. 2C). No dNFs were formed at pH 10 (Fig. 2D) and above (data not shown here) due to the occurrence of negative repulsion between dopamine molecules and PO_4_^3−^ ions.

**Figure 3:**
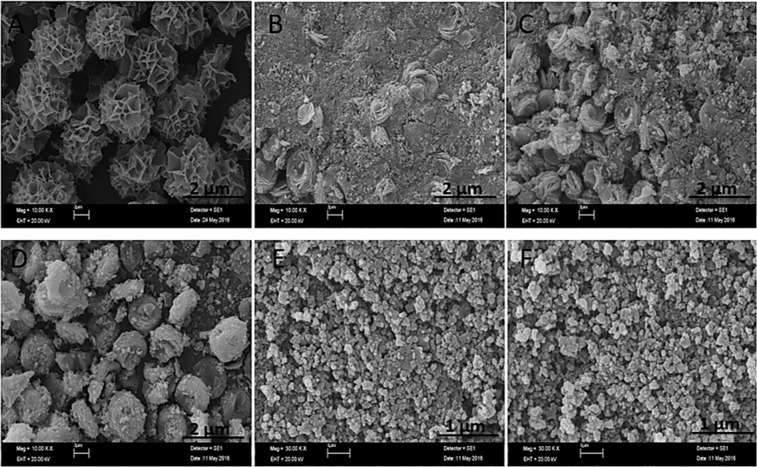
SEM images of dNFs using different concentrations of dopamine. (**A**) 0.01 mg/mL dopamine with 72 hrs incubation time, (**B–D**) 0.1 mg/mL dopamine with 24 hrs, 48 hrs and 72 hrs incubation times, respectively, (**E,F**) 0.5 mg/mL dopamine with 24 hrs and 72 hrs respectively.

**Figure 4:**
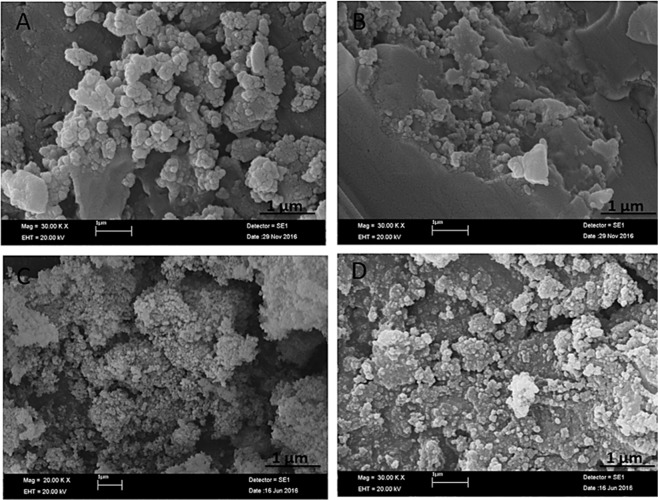
SEM images of dNFs using (**A**) Ni^2+^, (**B**) Zn^2+^, (**C**) Fe^2+^ and (**D**) Fe^3+^.

The optimal dopamine molecule concentration was determined in formation of ideal dNFs. While uniform dNF was produced in 72 hrs using 0.01 mg/mL (Fig. 3A), the increase in concentration of dopamine molecule delayed or prevented formation of dNFs. For instance, when 0.1 mg/mL dopamine was used, initial dNFs were seen in 24 hrs and 48 hrs as shown in Fig. 3B,C, respectively. Even after 72 hrs incubation, dNFs were produced with low yield (Fig. 3D). 0.5 mg/mL dopamine was incubated with Cu^2+^ ions in PBS solution for 24 hrs and 72 hrs, but no dNFs were formed as presented in Fig. 3E,F, respectively. Additionally, we examined how metal ions influence the formation of dNFs. It was expected to create dopamine and Ni^2+^, Zn^2+^, Fe^2+^, Fe^3+^ incorporated hybrid primary crystals for dNF formation, but those metals ions did not give suitable coordination reaction with amine group of dopamine, then no dopamine-Ni^2+^, -Zn^2+^, -Fe^2+^, -Fe^3+^ petals were formed. However, dNFs formations were succeeded as SEM images demonstrated in Fig. 4A–D, respectively.

**Figure 5:**
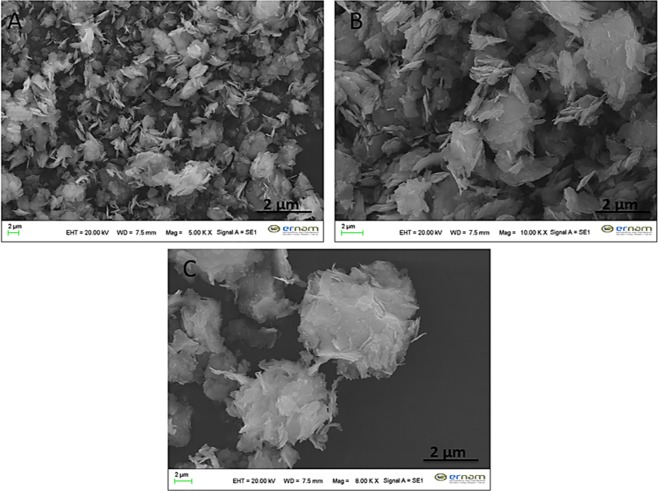
SEM images of dNFs by addition of dopamine on Cu_3_(PO_4_)_2_ primary crystals formed in different incubation time. (**A**) Cu_3_(PO_4_)_2_ formed in 1 day, (**B**) Cu_3_(PO_4_)_2_ formed in 2 days and (**C**) Cu_3_(PO_4_)_2_ formed in 3 days.

An interesting strategy called “post modification or post incubation” was tested for formation of dNFs. In typical NF synthesis, organic and inorganic components are simultaneously mixed into PBS solution for *in situ* synthesis of organic-inorganic hybrid NF. In contrast to that, dopamine molecules were added into pre-formed Cu_3_(PO_4_)_2_ primary crystals for synthesis of the dNFs. Basically, Cu^2+^ solution was added to PBS solutions (10 mM, pH 7.4) (final Cu^2+^ concentration was adjusted 0.8 mM) and Cu_3_(PO_4_)_2_ primary crystals with different morphologies were formed after 24 hrs, 48 hrs and 72 hrs incubation. 0.02 mg/mL dopamine was injected to each pre-formed Cu_3_(PO_4_)_2_ primary crystals solution, and then each resulting mixture was incubated 24 hrs for production of the dNFs. Unfortunately, the dNFs with ideal flower shaped morphologies were not obtained as shown in Fig. 5A–C compared to *in situ* NF synthesis method.

**Figure 6:**
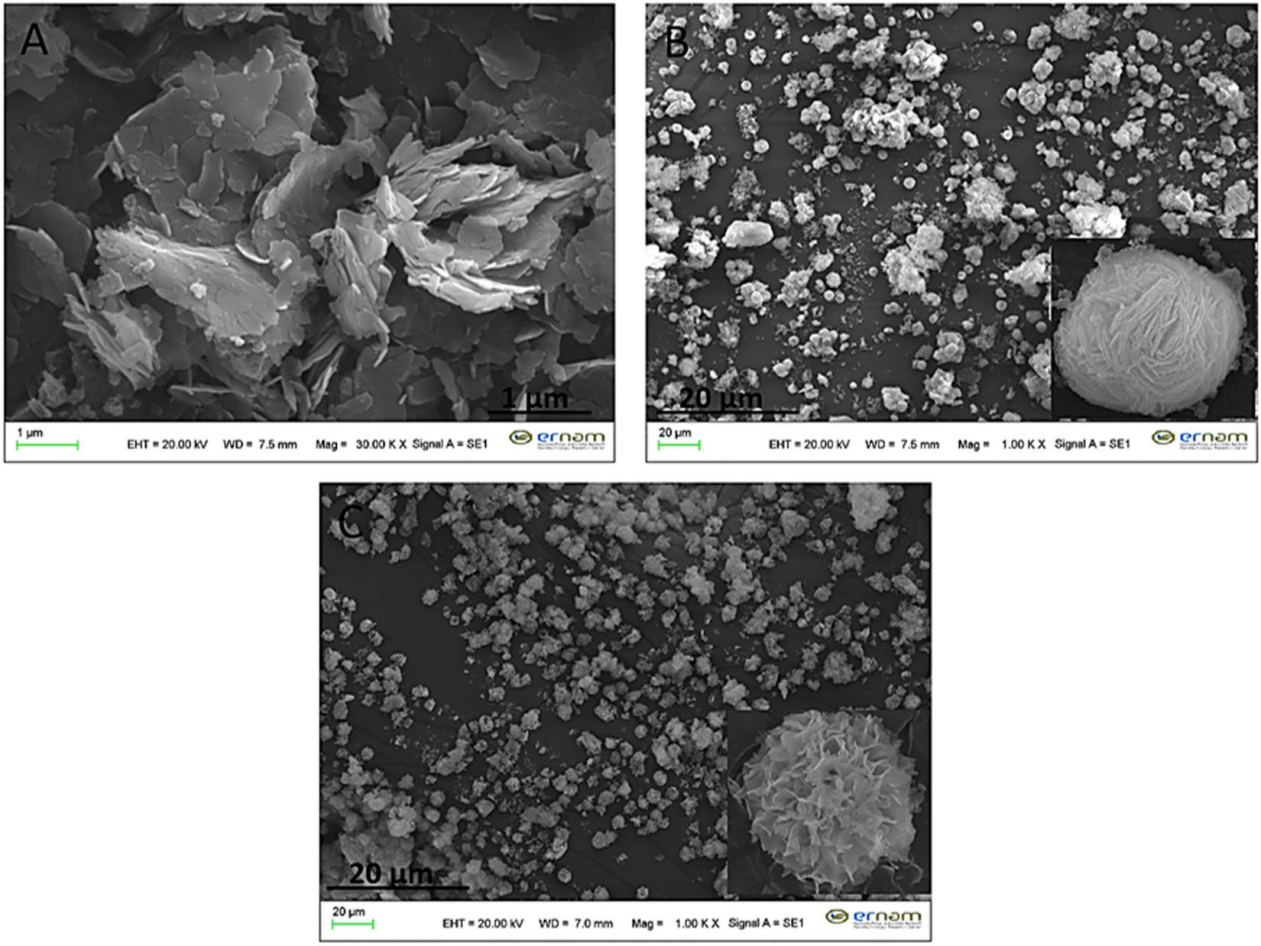
(**A**) Addition of EDTA on dNF, (**B**) formaldehyde treated dopamine molecule for dNF and (**C**) addition of formaldehyde on dNF.

In terms of stability of the dNFs, effects of ethylenediaminetetraacetic acid (EDTA) (used as a strong and versatile chelating agent forming complexes with transition-metal or heavy metal ions) and formaldehyde (acted as a crosslinking agent for biomolecules by reacting amine groups) were separately investigated on morphology of the dNFs. Figure 6A shows that Cu^2+^ ions were removed from the dNFs when treated with EDTA and flower shaped structure was collapsed. The formaldehyde and dopamine molecules were simultaneously added in PBS solution containing Cu^2+^ ions and incubated to show how formaldehyde influence formation of the dNF *in situ* synthesis (Fig. 6B) and pre-synthesized dNF solution was treated with formaldehyde (Fig. 6C). In both cases, SEM images presented that flower shaped structures were partially distorted and no full shape loss or no collapse were observed in Fig. 6B,C, respectively.

**Figure 7:**
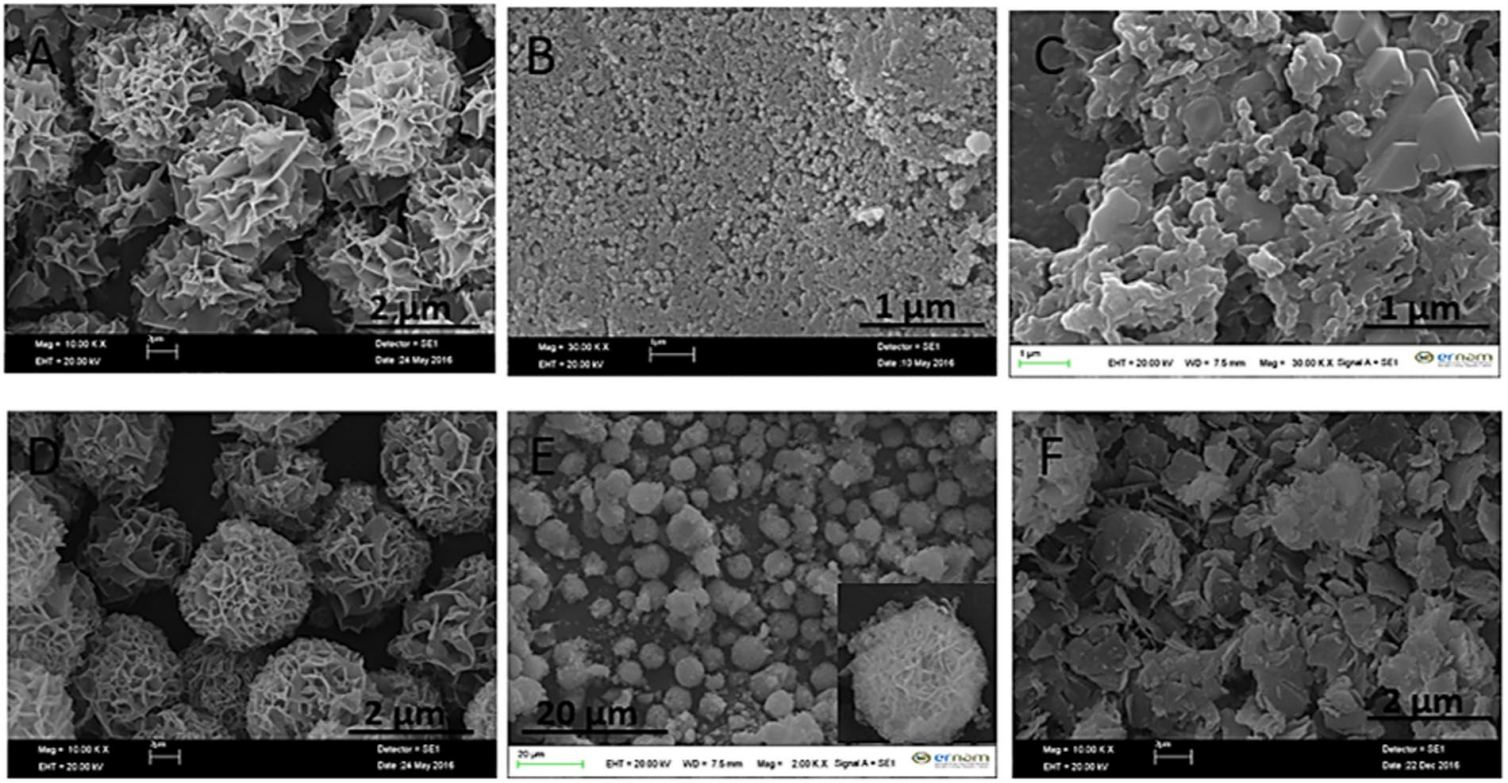
SEM images of epNFs using different concentrations of epinephrine. (**A–C**) 0.02 mg/mL, 0.1 mg/mL and 0.5 mg/mL. SEM images of neNFs using different concentrations of norepinephrine. (**D–F**) 0.02 mg/mL, 0.1 mg/mL and 0.5 mg/mL. Inlet: Magnified SEM image of neNF formed using 0.1 mg/mL norepinephrine given in (**E**).

In addition to the dNFs, epinephrine and norepinephrine as dopamine derivatives with different concentrations were utilized as organic parts for synthesis of the NFs. While 0.02 mg/mL epinephrine gave flower shaped epinephrine nanoflower (epNF) (Fig. 7A), no epNFs formations were succeeded with use of 0.1 mg/mL and 0.5 mg/mL epinephrine molecules as shown in Fig. 7B,C, respectively. The norepinephrine nanoflowers (neNFs) were successful produced using 0.02 mg/mL and 0.1 mg/mL norepinephrine as seen in SEM images of Fig. 7D,E. The morphology of neNF formed using 0.1 mg/mL norepinephrine was partially distorted due to repulsion of positively charged norepinephrine molecules. However, increasing norepinephrine concentration to 0.5 mg/mL resulted in formation of large petals, but no flower shaped structure could be obtained owing to lack of binding the petals together (Fig. 7F).

**Figure 8:**
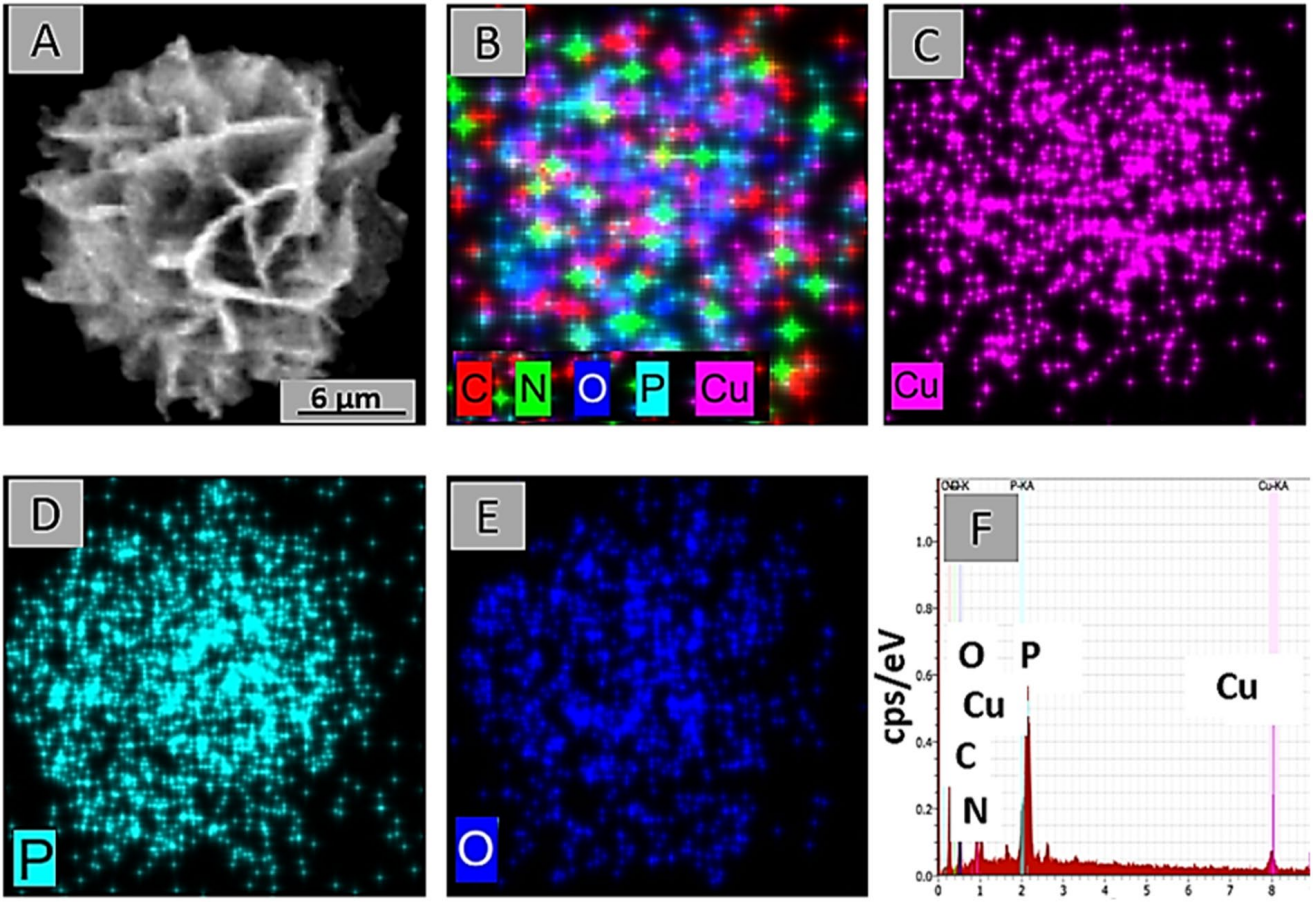
(**A–E**) Elemental mapping of dNF. (**F**) EDX analysis of dNF for presence of Cu metal.

**Figure 9:**
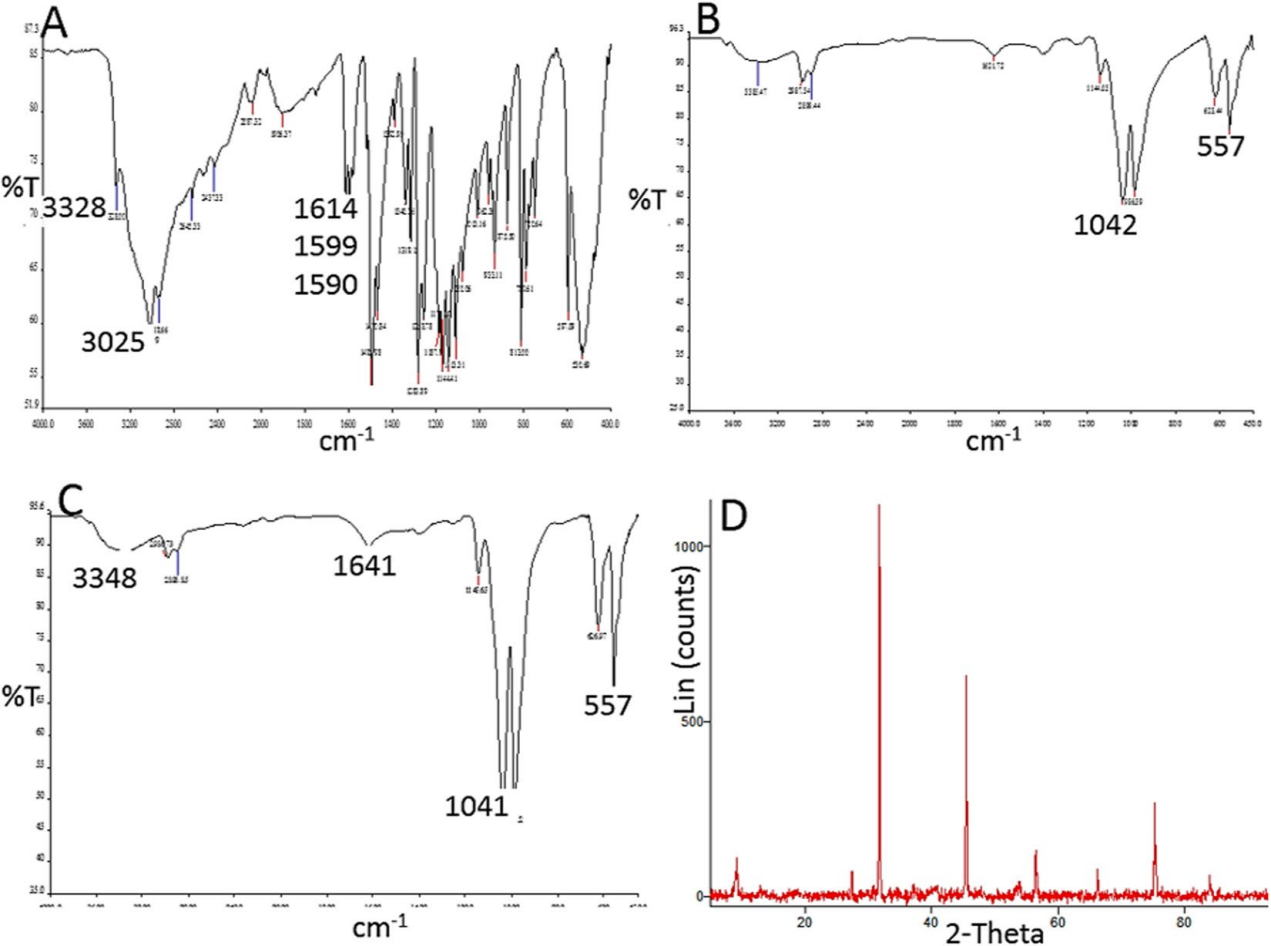
FTIR spectra of (**A**) free dopamine, (**B**) Cu_3_(PO_4_)_2_ primary crystal and (**C**) dNF. (**D**) XRD analysis of dNF.

**Figure 10:**
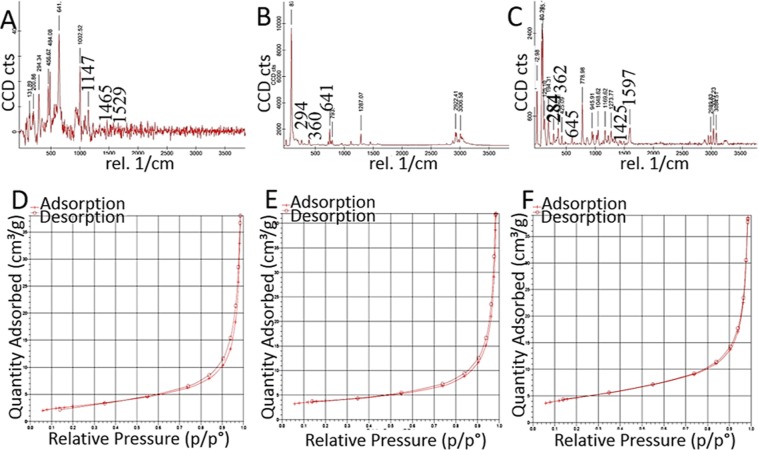
Raman spectra of (**A**) free dopamine, (**B**) Cu_3_(PO_4_)_2_ primary crystal and (**C**) dNF. BET isotherms of (**D–F**) dNF, epNF and neNF, respectively.

The dNFs as model NFs were further characterized with EDX analysis, mapping, FTIR, XRD, Raman and BET methods. The EDX mapping of the dNF (Fig. 8A) revealed presence of five different element including C, N, O, P and Cu in a merged image (Fig. 8B) and three main elements like Cu, P and O were demonstrated in separate images (Fig. 8C–E). The presence of the Cu metal as acted as a corner stone in the dNF formation was analysed with EDX spectrum (Fig. 8F). In terms of analysis of the dNF structure, Fig. 9A–C revealed bonds stretching and bending in FTIR spectra for free dopamine molecule, Cu_3_(PO_4_)_2_ primary crystal, and the dNF. The crystal structure of dNF with XRD analysis was also presented in Fig. 9D. Free dopamine molecules exhibited various characteristic stretching and bending vibrations in Fig. 9A. For instance, the bending and stretching bond for amine group can be attributed to 1614 cm^−1^ and 1599 cm^−1^, respectively. The diol groups of catecholamines gave stretching vibration at 3328 cm^−1^. C–C and C–H bonds of the aromatic ring resulted in stretching vibrations appeared at 1590 cm^−1^ and 3025 cm^−1^, respectively. The vibration peaks of PO_4_^3−^ in Cu_3_(PO_4_)_2_ primary crystals were observed at 1042 cm^−1^ and 557 cm^−1^ (Fig. 9B). FTIR spectrum of the dNFs shows in Fig. 9C that the bending amine bonds was assigned to 1621 cm^−1^ with left shift. The stretching vibration of diol groups of dopamine in the dNFs was appeared by shifting to 3348 cm^−1^. The corresponding peaks for vibrations PO_4_^3−^ were clearly seen at 1041 cm^−1^ and 557 cm^−1^. The peaks in FTIR spectrum of the dNFs indicate the successful incorporation of dopamine and Cu_3_(PO_4_)_2_ primary crystal in the dNFs. The crystal structure of the dNFs in Fig. 9D revealed diffraction peaks Cu_3_(PO_4_)_2_ primary crystal. Raman spectra of Cu_3_(PO_4_)_2_ primary crystal, free dopamine and dNF were used as complementary data to their FTIR spectra to prove the presence of dopamine and Cu_3_(PO_4_)_2_ in the dNF. For instance, the stretching peaks of aromatic rings in free dopamine were appeared at between distinct peak at around 1147 cm^−1^, weak peaks of 1410–1465 cm^−1^ and 1529 cm^−1^ (Fig. 10A). The characteristic stretching peaks of Cu-O bond in Cu_3_(PO_4_)_2_ were appeared with strong at around 294 cm^−1^, with weak peak 360 cm^−1^ and with very strong peak at 641 cm^−1^ (Fig. 10B). Additionally, the aromatic rings of dopamine in the dNF gave stretching peaks with weak and strong intensity at around 1425 cm^−1^ and 1597 cm^−1^, respectively (Fig. 10C). The peaks with different intensities at 284 cm^−1^, 362 cm^−1^ and 645 cm^−1^ were ascribed to characteristic stretching peaks of Cu-O bonds in the dNF (Fig. 10C). BET method relied on nitrogen adsorption-desorption measurement was utilized to determine the surface area of dNF, epNF and neNF shown in Fig. 10D–F, respectively. The narrow hysteresis loop of each NF was in the range of 0.8–1.0 P/P_0_, which can be considered as indication of as a type IV isotherm^22^. While single point surface areas at P/Po (6.3195 m²/g and 9.1179 m²/g) and BET surface areas (4.0867 m²/g and 6.4919 m²/g) were measured for dNF (Fig. 10D) and epNF (Fig. 10E), even much higher single point surface area (24.5823 m²/g) and BET surface area (13.1667 m²/g) were obtained with neNF (Fig. 10F).

**Figure 11:**
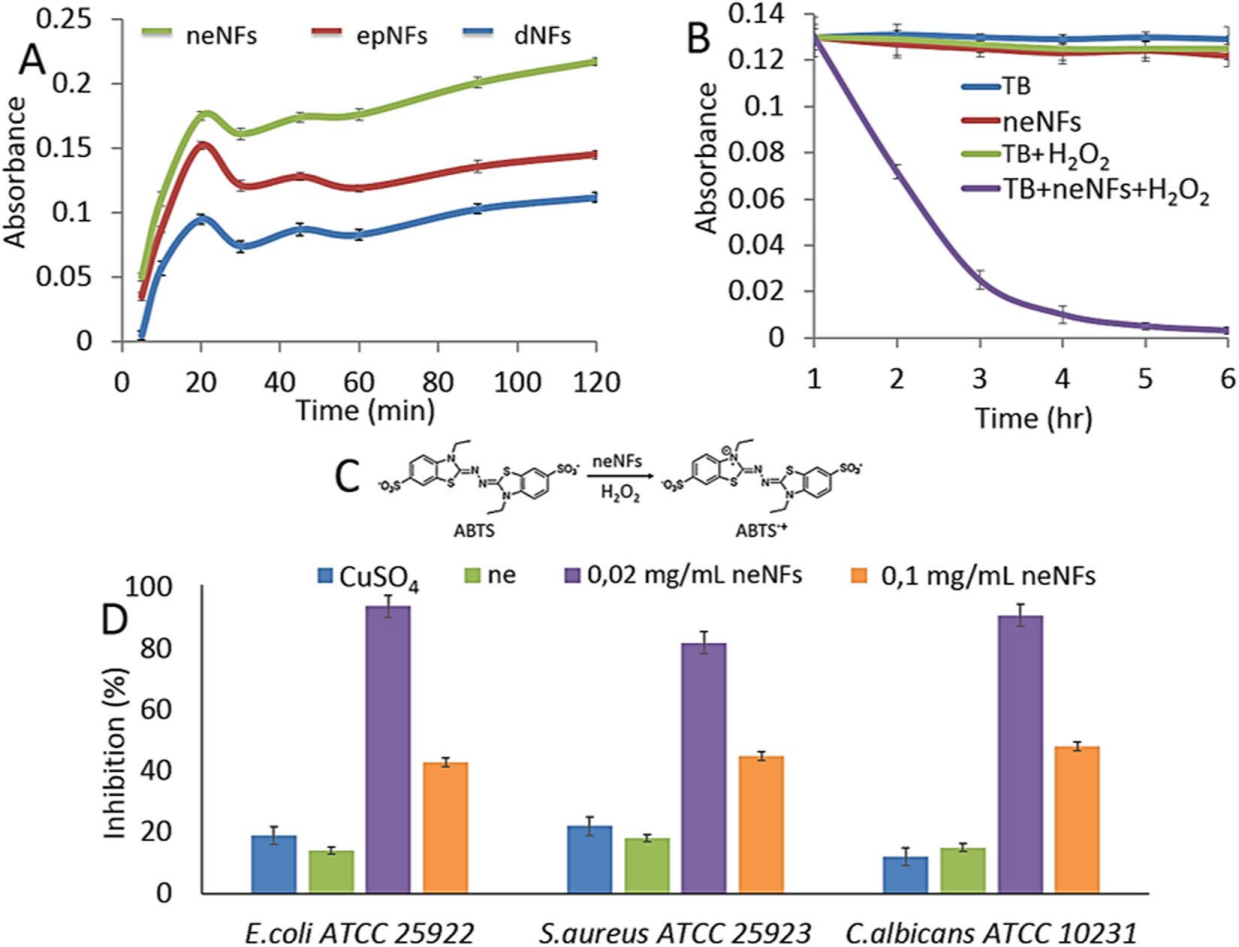
(**A**) Peroxidase like activity of cNFs, (**B**) dye degradation (thiazolyl blue, TB) activity of neNF and (**C**) The reaction for oxidation of ABTS into the radical cation ABTS^•+^. (**D**) Antimicrobial activities of CuSO_4_, norepinephrine and neNFs.

The intrinsic peroxidase-mimic activities of dNF, epNF and neNF were systematically tested towards ABTS for catalytic activities, thiazolyl blue tetrazolium bromide for dye degradation activities and microorganisms (*E. coli* ATCC 35218, *S. aureus* ATCC 25923 and *C. albicans* ATCC 10231) for antimicrobial activities as presented in Fig. 11A–C. Benefiting from function of NFs as a Fenton reagent allows us to use them against these substrate, organic dye and microorganisms. In principle, copper compounds exhibit peroxidase-like activities through Fenton reaction in the presence of hydrogen peroxide (H_2_O_2_) by catalysing the oxidation of proton-donor compounds. However, the catalytic activity of copper ions or copper compounds is very low and dependent on some experimental parameters such as temperature and pH and so like. To address these issues, our results showed that copper ions coordinated in the NFs exhibited quite much catalytic activities compare to free transition metal ions and copper crystals^21,32^. The potential mechanism for Fenton-like reaction of the NFs can be demonstrated in Eq. 1 (Eq. 1) with following step: (i) Cu^2+^ ions in NFs react with hydrogen peroxide to form Cu^1+^ ions, (ii) as a result of the reaction between of Cu^1+^ and H_2_O_2_, highly reactive hydroxyl radical is formed, (iii) and this free hydroxyl radical causes substrate oxidation.

1 $$\begin{array}{c}{{\rm{Cu}}}^{2+}+{{\rm{H}}}_{2}{{\rm{O}}}_{2}\to {{\rm{Cu}}}^{+}+{{\rm{HOO}}}^{\cdot }+{{\rm{H}}}^{+}\\ {{\rm{Cu}}}^{+}+{{\rm{H}}}_{2}{{\rm{O}}}_{2}\to {{\rm{Cu}}}^{2+}+{{\rm{HO}}}^{\cdot }+{{\rm{OH}}}^{-}\end{array}$$

The peroxidase mimic activities of the dNF (blue line), epNF (red line), and neNF (green line), were performed towards ABTS. The rapid and effective oxidation of ABTS into the radical cation ABTS^•+^ was spectrophotometrically monitored via absorbance of the product (ABTS^•+^) at 417 nm. We demonstrated that one-electron the oxidative activities of the neNF were much higher than that of dNF (blue line), epNF (red line) due to high surface area (consistent with BET results) and much polar surface properties of neNF. We also used neNF for dye degradation and antimicrobial activities owing to their quite high peroxidase mimic activities compare to other cNFs. The neNF acted as a promising alternative to enzymes for effectively remove the thiazole blue tetrazolium bromide dye presence of H_2_O_2_. The Fig. 11B demonstrated that the dye was drastically and almost completely decomposed within 3 hrs incubation. No decomposition was observed when only thiazole blue  tetrazolium bromide itself (blue line), thiazole blue tetrazolium bromide + neNFs (red line), and thiazole blue tetrazolium bromide + H_2_O_2_ (green line). The results stated that function of neNF as a Fenton reagent plays a crucial role for dye decomposition by generating Cu^1+^ ions and highly reactive hydroxyl radicals in the presence of H_2_O_2_, which resulted in oxidation or quenching of the dye.

The antimicrobial activities of CuSO_4_, free norepinephrine and neNFs were investigated in Fig. 11D. The 0.8 mM CuSO_4_ in the presence of H_2_O_2_ displayed low antimicrobial activities by ∼19%, ∼22% and ∼12% *E. coli*, *S. aureus* and *C. albicans* cell inactivations, respectively (blue bar in Fig. 11D). Similar to that, 1 mg/mL of free norepinephrine acted as a mild antimicrobial agent and resulted in ∼14%, ∼18% and ∼15% inactivation for *E. coli*, *S. aureus* and *C. albicans*, respectively (green bar in Fig. 11D). In the presence of H_2_O_2_, while 1 mg/mL neNF killed (formed using 0.02 mg/mL norepinephrine) ∼94%, ∼82% and ∼91% of *E. coli*, *S. aureus* and *C. albicans* cells (purple bar in Fig. 11D), the decrease in antibacterial activity was observed when using same amount of the neNF (formed using 0.1 mg/mL norepinephrine), which killed ∼43% of *E. coli*, ∼45% of *S. aureus* and ∼48% of *C. albicans* cells (orange bar in Fig. 11D). We interpret that CuSO_4_ exhibited quite low Fenton reaction compared to the neNFs. Interestingly, the neNF (formed using 0.1 mg/mL norepinephrine) showed less antimicrobial activity compared to the neNF (formed using 0.02 mg/mL norepinephrine) owing to rapid of aggregation of the neNFs formed from 0.1 mg/mL norepinephrine which may prevent effective reactive hydroxyl radical production and interaction between the neNF and microorganisms. We claim that, distorted morphology of the neNF may negatively affect antimicrobial activities of the neNFs (formed from 0.1 mg/mL).

## Conclusions

We have systematically examined formation of catecholamines (dopamine, epinephrine and norepinephrine)-copper ion (Cu^2+^) incorporated flower shaped hybrid nanostructures as function of catecholamine concentrations, incubation time and pH values of PBS solutions. Among the catecholamines nanoflowers (cNFs), dopamine and norepinephrine nanoflowers (dNF and neNF) were almost formed in 3 hrs incubation owing to their structural similarities, but epinephrine nanoflower (epNF) was obtained in 24 hrs. We demonstrated that dNF as a model catecholamine was not form at pH 5 and below and at pH 10 and above due to positive and negative repulsions, respectively. In terms of catecholamine concentrations, while dNF and epNF were not formed at 0.1 mg/mL and above, neNF was successfully synthesized using only 0.1 mg/mL norepinephrine but not above. We experimentally proved that neNF acted as a much effective Fenton agent among other cNFs. The neNF exhibited much higher peroxidase-mimic catalytic, dye degradation and antimicrobial activities compared to dNF and epNF owing to its porous structure, high surface area and polar surface property. Finally, we propose that catecholamines nanoflowers (cNFs) with their intrinsic peroxidase like activities can be used where enzyme nanoflowers have been utilized.
